# Potent optogenetic regulation of gene expression in mammalian cells for bioproduction and basic research

**DOI:** 10.1093/nar/gkaf546

**Published:** 2025-06-30

**Authors:** Jeannette Gebel, Elisa Ciglieri, Rainer Stahl, Fraser Duthie, Fabian Frechen, Andreas Möglich, Herbert Müller-Hartmann, Hanns-Martin Schmidt, Dagmar Wachten

**Affiliations:** Institute of Innate Immunity, Biophysical Imaging, Medical Faculty, University of Bonn, 53127 Bonn, Germany; Ningaloo Biosystems GmbH, 51011 Cologne, Germany; Institute of Innate Immunity, Innate Immunity & Metaflammation, Medical Faculty, University of Bonn, 53127 Bonn, Germany; Institute of Innate Immunity, Innate Immunity & Metaflammation, Medical Faculty, University of Bonn, 53127 Bonn, Germany; Institute of Innate Immunity, Biophysical Imaging, Medical Faculty, University of Bonn, 53127 Bonn, Germany; Department of Biochemistry, University of Bayreuth, 95447Bayreuth, Germany; Ningaloo Biosystems GmbH, 51011 Cologne, Germany; Ningaloo Biosystems GmbH, 51011 Cologne, Germany; Institute of Innate Immunity, Biophysical Imaging, Medical Faculty, University of Bonn, 53127 Bonn, Germany

## Abstract

Precise temporal and spatial control of gene expression greatly benefits the study of specific cellular circuits and activities. Compared to chemical inducers, light-dependent control of gene expression by optogenetics achieves a higher spatial and temporal resolution. Beyond basic research, this could also prove decisive for manufacturing difficult-to-express proteins in pharmaceutical bioproduction. However, current optogenetic gene-expression systems limit this application in mammalian cells, as expression levels and the degree of induction upon light stimulation are insufficient. To overcome this limitation, we designed a photoswitch by fusing the blue light-activated light–oxygen–voltage receptor EL222 from *Erythrobacter litoralis* to the three transcriptional activator domains VP64, p65, and Rta in tandem. The resultant photoswitch, dubbed DEL-VPR, allows up to a 570-fold induction of target gene expression by blue light, thereby achieving expression levels of strong constitutive promoters. Here, we used DEL-VPR to enable light-induced expression of complex monoclonal and bispecific antibodies with reduced byproduct expression and increased yield of functional protein complexes. Our approach offers temporally controlled yet strong gene expression and applies to academic and industrial settings.

## Graphical Abstract

**Graphical Abstract:**
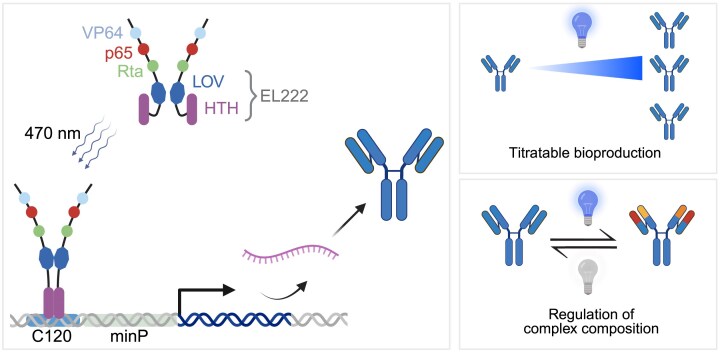

## Introduction

Biopharmaceuticals, such as monoclonal (mAb) and bispecific (bsAb) antibodies, are increasingly in demand as therapies for difficult-to-treat afflictions like cancer by promoting, e.g. the specific recruitment of T or NK cells to cancerous cells [1]. While biopharmaceuticals offer many advantages, such as reducing off-target and side effects, challenges remain. These include, first and foremost, high production costs, primarily attributable to low yields, impurities, and limited scope for automation.

In contrast to classic IgG antibodies, bsAbs do not possess two identical antigen-binding sites but two different ones. However, the production of bsAb with two distinct light (LC) and heavy (HC) chains leads to high levels of undesired by-products (e.g. 2 LC + 2 HC with the same antigen binding site) [2, 3]. Hence, single-chain variable fragments (scFvs) are often used, containing the LC and HC variable fragments. Together with other technologies, such as knobs-into-hole mutations [4], mispairing in bsAb production can be reduced but not completely prevented. Static or temporal imbalances in the single-chain production contribute to the high costs of bsAb.

One avenue towards overcoming these restrictions is the precise timing and modulation of gene expression to improve protein production, quality, and yield. Chemical induction tools, such as tetracycline- or cumate-based systems, are commonly used in research and production [5–7]. However, these systems are costly, affect cell viability, and lack dynamic, temporal, and spatial resolution.

Current challenges in gene induction could be resolved through optogenetic regulation of gene expression. Optogenetics enables the control of diverse cellular signaling processes, e.g. membrane potential, cellular signaling pathways, and gene expression by light [8–11]. Systems regulated by blue light show high versatility, efficiency, and favorable kinetics [12]. Notably, blue-light-responsive photoswitches have been advanced for regulating gene expression in a variety of organisms, spanning bacteria [13–16], yeast [17], and mammals [18, 19]. Setups for the optogenetic induction of gene expression generally rely on light-activated protein–protein and protein-DNA interactions [12, 20]. In mammalian cells, blue light-activated optogenetic systems are based on either plant cryptochromes [21, 22] or light–oxygen–voltage (LOV) domains [23–25]. Although phytochrome-based systems have been used for optogenetic gene expression control in eukaryotic cells [26], despite recent efforts [27], they require reduced bilin chromophores, which are absent in mammals. In contrast, cryptochromes and LOV receptors resort to flavin-nucleotide chromophores, ubiquitously abundant as redox-active cofactors in mammalian cells. Compared to cryptochrome- and phytochrome-based systems, which generally need at least two different components for photosensing, certain LOV receptors rely on a single polypeptide component and act by forming a light-induced homodimer [26, 28, 29]. This makes the system less prone to variations in transfection efficiency and cellular expression levels. Moreover, it reduces the genetic footprint of the optogenetic circuit and saves valuable space in expression vectors, especially when using viral vectors.

The LOV protein EL222, deriving from *Erythrobacter litoralis* HTCC2594 [30], consists of two functional domains, the light-sensitive LOV domain and a helix–turn–helix (HTH) DNA-binding domain. In the resting state, the pivotal HTH 4α helix is covered by the LOV domain, which prevents dimerization and DNA binding [30]. Blue light induces the formation of a metastable covalent adduct between a cysteine residue of the LOV domain and flavin mononucleotide, which serves as the light-sensing cofactor [24, 31, 32]. The concomitant protonation of the flavin N5 atom triggers conformational changes [33] and, thus, releases the HTH domain, which leads to receptor dimerization and DNA binding of the HTH domain [34, 35]. To achieve an optimized DNA binding of EL222, the so-called Clone 1–20 bp (C120) sequence with a downstream TATA box promoter is often used for gene induction [29, 34]. The LOV system has activation kinetics of only a few seconds with a spontaneous reversion in the dark (ca. 50 s at ambient temperature) [29, 34]. In previous studies, EL222 only showed minor basal activity of noninduced gene expression combined with limited cytotoxicity [29, 34, 36].

Modular photoswitches have been developed using EL222 as the sensing domain, of which two, VP-EL222 and VP-EL222-NLS (VEL), are particularly attractive [29, 37]. Both tools incorporate the activation domain VP16 from the herpes simplex virus (HSV) 1. VP-EL222 is characterized by one nuclear localization sequence (NLS) [29], while VEL, which features an optimized EL222 sequence, contains two NLS. However, VP-EL222 failed to induce detectable mCherry expression in HEK293 cells [38], a standard bioproduction cell line. Accordingly, to enhance the light-mediated regulation to produce biotherapeutic antibodies in mammalian cell lines, we extended VP16 to VPR as transactivation domain [39–41]. VPR is a fusion product of VP64 (four repeats of the VP16), the transactivation domains of the NF-kappa B subunit p65 [42], and the Epstein-Barr virus R transactivator (Rta) [39]. The combined action of these three components results in synergistic enhancement of transcriptional induction and has been widely used for artificial induction, e.g. in the context of CRISPR activation [40, 41, 43]. Hence, EL222 fused to VPR, called DEL-VPR, is a promising candidate for a powerful and dynamic regulation of bioproduction processes.

In this study, we show that DEL-VPR supports a strong induction of protein expression in HEK293T and CHO-K1 cells under blue light, reaching levels of cytomegalovirus (CMV) promoter-driven constitutive protein expression combined with a low basal activity. These favorable traits allowed the expression of biotherapeutics such as monoclonal and bispecific antibodies with high yield and purity.

## Materials and methods

### Plasmids

The VP-EL222 was synthesized (Integrated DNA Technology) based on the sequence from Motta Mena *et al.* [29]. The VEL construct and VPR sequence were synthesized by GeneArt (Thermo Fisher). VP-EL222 and VEL were singularly cloned inside a pcDNA3.1 vector (Invitrogen) using *BamHI* and *XbaI* restriction sites. For the light-induced expression of firefly luciferase (fLuc), the constructs pGL4.23_5xC120-minP-FLuc and pcDNA3.1_5xC120-minP-FLuc were generously provided by Kevin Gardner. The plasmids encoding GAVTA and TAEL were generously provided by Stephanie Woo. The construct 5xC120-minP-mCherry, used for light-induced expression of the fluorescent reporter mCherry, was created by cloning polymerase chain reaction (PCR)-amplified mCherry into pcDNA3.1_5xC120-minP-FLuc vector using *EcoRI* and *XbaI* restriction sites. Primers are described in the Supplementary Table S1.

For adeno-associated virus (AAV) production and the assessment of mScarlet3 expression, C120-mScarlet3 and CMV-DEL-VPR were cloned into pAAV2 using *SacI/EcoRI* and *HindIII/XbaI*, respectively. CMV-mScarlet3 was cloned into pAAV2 using *SacI/EcoRI*.

Human IFNb was cloned into a self-made vector either behind the C120 or CMV-promoter using *DraII/ClaI*. CMV-DEL-VPR was cloned into the C120-IFNb containing construct using *HindIII/XbaI*.

As a model for monoclonal IgG antibody (mAb) expression, we chose the tetrameric trastuzumab/Herceptin, consisting of two LCs and two HCs, which was generously provided by Andrew Beavil (Addgene, #61883). For the light-induced expression, the mAb chains were amplified via PCR and subcloned into pcDNA3.1_5xC120-minP-FLuc vector using *AscI* and *MreI* restriction sites. For the constitutive expression, the mAb chains were PCR-amplified and subcloned into pcDNA3.1 under the control of the CMV promoter, utilizing either *AscI* and *XhoI* or *AscI* and *MreI* restriction sites.

The plasmid encoding the bsAb was generously provided by Lonza. The expressed protein is a trimeric IgG bsAb with an LC and HC on one side and a single-chain variable chain (HCscFv) on the other (proprietary to Lonza). The formation of HC or HCscFv homodimers is limited by the knobs-into-holes technology. For constitutive expression, all three bsAb chains were encoded on the same plasmid, individually driven by a CMV promoter. For the light-induced expression of the different bsAb chains, the single-chain DNA sequences (LC, HC, and HCscFv) were synthesized by GeneArt (Thermo Fisher) and inserted into the pcDNA3.1_5xC120-minP vector ordered at Genscript. For the constitutive expression of the HCscFv, the synthesized sequence was inserted into a pInducer20 vector under the control of the H1 promoter.

### Cell lines

HEK293T and CHO-K1 were obtained and authenticated from the American Type Culture Collection. HEKblue IFN reporter cells were obtained from Invivogen.

HEK293T and HEKblue IFN reporter cells were maintained in Dulbecco’s modified Eagle’s medium (DMEM) (Gibco, #10564011) and 10% fetal calf serum (FCS) (Biochrome) at 37°C and 5% CO_2_. CHO-K1 cells were maintained in DMEM/F12 1:1 (Gibco, #11320033) with 10% FCS at 37°C and 5% CO_2_.

### Transfection

For the luciferase assays and the live-cell monitoring, 30,000 HEK293T cells/well were seeded in DMEM + 10% FCS in two 96-well plates. One plate was exposed to blue light, whereas the other plate was kept in the dark. One hour after seeding, cells were transfected with 0.1 μg DNA/well using Lipofectamine 3000 (Invitrogen, #L3000001) in a ratio of 1:3 (DNA:Lipofectamine). For the luciferase assays, the following genes were transfected in the ratio 5:1:0.1: blue light-sensitive photoswitches. (VP-EL222, VEL, DEL-VPR, GAVTA, GAVPO, or TAEL), a light-sensitive luminescent reporter [(C120)_5_-fLuc for VP-EL222, VEL, DEL-VPR and TAEL or UAS-fLuc for GAVTA and GAVPO], and constitutive luminescent reporters (SV40-renilla luciferase for the normalization of the firefly measures and HSV TK/SV40/CMV/H1-fLuc as references for constitutive protein expression).

For live-cell monitoring, the following genes were transfected in the ratio 5:1: (i) blue-sensitive photoswitches (VP-EL222, VEL, or DEL-VPR) and (ii) light-sensitive fluorescent reporter or constitutive reporter [(C120)_5_-mCherry or CMV/H1-mCherry]. Cells were transfected 8 h before light induction.

For antibody expression, cells were seeded at 140,000 cells/well in a 24-well plate (Sarstedt) 5 h before transfection in OptiMEM (Gibco, #11058021). HEK293T cells were transfected with 500 ng DNA using PEI (Sigma, #408727) in a DNA to polyethylenimine (PEI) ratio of 1:2. CHO-K1 cells were transfected with 500 ng DNA using Lipofectamine 3000. For mAb experiments, the DEL-VPR, C120-LC, and C120-HC-encoding plasmids were transfected in a ratio of 3:1:1. For the bsAb experiments, the DEL-VPR, C120-LC, C120-HC, and C120-HCscFv-encoding plasmids were transfected in a ratio of 3:1:1:1. The cells were transfected 24 h before light induction.

### Stable cell lines

For the generation of stable HEK293T lines, cells were transfected as described above with CMV-driven GS piggyBac® transposase and the plasmid containing the gene of interest (GOI) in a molar ratio of 1:1 using PEI. The GOI plasmids for constitutive or light-dependent expression contained either three independent CMV or C120 promoters, respectively, in front of the three bsAb chains on a single construct. The GOI plasmid for light-dependent regulation contained a CMV-flox-mCherrySTOP-flox-DEL-VPR. The GOI was flanked with inverted terminal repeats (ITR) for integration. The integrated fragment contained a hygromycin resistance gene. The cells were cultivated after transfection in HEK medium (DMEM + 10% FCS), and 24 h after transfection, hygromycin was added at 10 μg/ml to the medium. The cells were selected and expanded for 14 days.

Cells were seeded after the selection at 60,000 cells/well in a 96-well plate in HEK medium. For the activation of DEL-VPR, cells were treated with TAT-CRE (Merck, #SCR508) at 100 U/ml for 24 h. The medium was changed to OptiMEM afterward.

### Optogenetic stimulation

Cells were illuminated at 470 nm (always ON unless otherwise stated) at the indicated intensities (μW/cm^2^) for the indicated duration using the MLA-1 multiformat illumination device (Ningaloo Biosystems) at 37°C and 5% CO_2_. The dark controls were always kept in the dark.

### Luciferase assay

Cells were excited with constant blue light (470 nm) for 9 h, starting 8 h after transfection, and a light intensity of 1500 μW/cm^2^. After illumination, the dual luciferase assay was performed using the Dual-Glo® kit (Promega, #E2940), according to the manufacturer’s protocol. Briefly, the culture medium was removed and substituted with 40 μl/well Opti-MEM, followed by the addition of 40 μl Dual-Glo® Reagent and incubation for 30 min to promote cell lysis. Afterward, the firefly luminescence was measured with a FLUOstar Omega microplate reader (BMG). Next, the firefly luminescence was quenched by adding 40 μl Dual-Glo® Stop & Glo® Reagent, followed by 30 min incubation, after which the renilla luminescence was measured. The ratio between the firefly and the renilla luminescence allowed for normalization of the measured values.

### Secreted alkaline phosphatase assay

Cells were transfected as described above with C120-IFNb and CMV-DEL-VPR on a single plasmid. Constitutive control cells were transfected with CMV-IFNb. After 24 h of transfection, the cells were illuminated at indicated intensities and pulses for 24 h. The supernatant was collected and added at 1% (v/v) onto 80,000 cells/well (96-well plate) HEK-IFNb Blue cells (Invivogen, # hkb-ifnabv2) for 24 h in HEK medium. The HEK-IFNb Blue cells express secreted alkaline phosphatase (SEAP) in response to IFNb. SEAP activity was determined by mixing 10 μl supernatant of the treated HEK-IFNb Blue cells with 90 μl of Quanti-Blue (Invivogen, #rep-qbs). The solution was incubated for 30 min at 37°C. The absorption was measured at 630 nm using a plate reader (Tecan Infinite 200 pro).

### Live-cell imaging

The light-exposed samples were excited with constant blue light (470 nm) for 8 h, starting 8 h after transfection, and a relative light intensity of 1500 μW/cm^2^. Then the samples were moved back and forth every 4 h for a period of 8 h between the blue light source and the IncuCyte reader (Sartorius) to allow the acquisition of the brightfield and the red channels. After 8 h, the samples were illuminated for an additional 16 h without image collection. Ultimately, the samples were placed back into the IncuCyte reader for 24 h, where pictures were collected again at intervals of 4 h. The dark samples were kept inside the IncuCyte for the entire experiment. The images collected with the IncuCyte reader were analyzed using the IncuCyte analyzer software (IncuCyte^®^ Base Analysis Software). Consistent image processing parameters were applied across all the experiments to both the phase channel, for measuring cell confluency, and to the red channel, for quantifying the number of fluorescent cells per mm^2^ and their integrated intensity (OCU × μm^2^/mm^2^).

### Flow cytometry

For flow cytometry, C120-mScarlet3 and CMV-DEL-VPR (encoded on the same plasmid) or CMV-mScarlet3 were transfected. After light induction, the cells were detached using trypsin (Gibco, #25200072), centrifuged at 500 × *g* for 5 min, and resuspended in phosphate-buffered saline (PBS). To assess the viability of the cells, Zombie Violet (BioLegend, #423113) was added at 0.1% (v/v) and incubated for 30 min on ice. The cells were centrifuged at 500 × *g* for 5 min and resuspended in flow buffer (PBS + 1% bovine serum albumin (BSA) + 1 mM ethylenediaminetetraacetic acid (EDTA)). Samples were analyzed using the Attune NxT flow cytometer (Invitrogen) and analyzed with FlowJo (version v10.10).

### Western blot

Cell culture supernatants were collected 24 h after light induction, and cellular debris was removed. A nonreducing loading dye (200 mM Tris/HCl, pH 6.8, 8% (w/v) SDS, 50% (v/v) glycerin, 0.04% bromophenol blue) was added to the samples for nonreducing western blots (WBs). Samples were denatured for 5 min at 95°C and loaded on precast 4%–20% sodium dodecyl sulfate–polyacrylamide gel electrophoresis (SDS–PAGE) (Biotrend, #DG101-02-V2). Proteins were blotted on PVDF membranes (Merck Millipore, #T381.1) using a semi-dry blotter (BioRad, #T788.1). Membranes were blocked in 5% BSA (Sigma–Aldrich, #A2153). Produced antibodies were detected with a goat-anti-human IgG antibody coupled to HRP (Thermo Fisher; 1:5000) and ECL (Perkin Elmer, #10400505). The band intensities were quantified using Fiji [44].

### ELISA

Crude cell culture supernatants were collected after 24 h of light induction. Antibody production was detected using the Enzyme-Linked Immunosorbent Assay (ELISA) human IgG total kit (Thermo Fisher, #88-50550-22) according to the manufacturer’s protocol. The absorption was measured using a plate reader (Tecan Infinite 200 pro).

To test the functionality of the mAb trastuzumab, the antigen HER2 (Biotechne, #AVI1129) and HER1 (Biotechne, #AVI10493) as negative control were coated at 5 μg/ml on a Strep-Tactin-coated plate (IBA, #2-5101-001) overnight at 4°C. The crude cell culture supernatants were collected after 24 h of light induction and incubated on the antigen-coated plates for 4 h at RT. For the rest of the procedure, the ELISA human IgG total kit (Thermo Fisher, #88–50550-22) was used according to the manufacturer's protocol. The absorption was measured using a plate reader (Tecan Infinite 200 Pro). The functionality of the bsAb was assessed in the same manner; however, due to the confidentiality of the provided bsAb (Lonza), we cannot disclose the identity of the antigens.

### AAV transduction of small-molecule neural precursor cells

The AAV 2.1 was produced by transfecting 800,000 HEK293T cells/well in a 6 cm dish with a plasmid containing the GOI (either CMV-mScarlet3 or C120-mScarlet3 + CMV-DEL-VPR) and AAV2.1 helper plasmids using PEI. The medium was changed 8 h after transfection. The supernatants of 6 wells/conditions were collected 72 h after transfection, combined, and precipitated in 8% PEG 8000 at 4°C for 6 h. The solution was centrifuged at 2,000 g and 4°C for 15 min. The pellet was resuspended in 300 μl PBS.

For testing the light-inducible promoter system in hiPSC-derived small molecule neural precursor cells (smNPCs) [45], 50,000 cells/well were seeded on a Geltrex (Thermo) coated, black cyclic olefin 96-well PhenoPlate (Revvity). For coating, plates were coated with Geltrex 1:100 in cold DMEM-F12 (Gibco). smNPCs were cultivated in N2B27 medium (Gibco) consisting of DMEM-F12/Neurobasal (Gibco) 50:50 with 1:200 N2 supplement (Gibco), 1:100 B27 supplement without vitamin A (Gibco), and 1% penicillin/streptomycin (Gibco), as well as 3 μM CHIR 99021 (Axon Medchem), 0.5 μM purmorphamine (Stem Cell), and 150 μM ascorbic acid (Sigma). After 24 h of cultivation, cells were transduced with freshly produced AAV either carrying the CMV-driven mScarlet3 reporter or the light-inducible DEL-VPR-driven mScarlet3 reporter. The AAV was diluted 1:10 in N2B27 media and kept on the cells for 48 h before the media was changed to fresh N2B27 media in the dark. For light stimulation, cells were either kept in the dark or exposed to blue light for 24 h. Cells were fixed with 4% paraformaldehyde (PFA) in PBS for 10 min at room temperature before being stained with DAPI 1:10,000 (Invitrogen). Images were acquired using the confocal SP8 microscope (Leica). Images were analyzed via ImageJ (version 1.53).

### Statistical analysis

Data were plotted using Prism (Version 10.4.2). Prism was also used to perform statistical analysis. Before determining the *P*-values between independent samples, the normality of their distribution was verified with the Shapiro-Wilk test. If passed, the Student’s *t*-test was performed; if not, the Mann–Whitney test. The one-way ANOVA followed by the Holm-Sidak *post-hoc* test was performed for comparing more than two groups with one independent variable, and for more than two groups with two independent variables, the two-way ANOVA with Holm-Sidak post-hoc test. The data is represented as mean ± SD of in the figure legend indicated number of biological independent repetitions.

## Results

### Design of the modular blue-light sensitive photoswitch DEL-VPR

**Figure 1.**
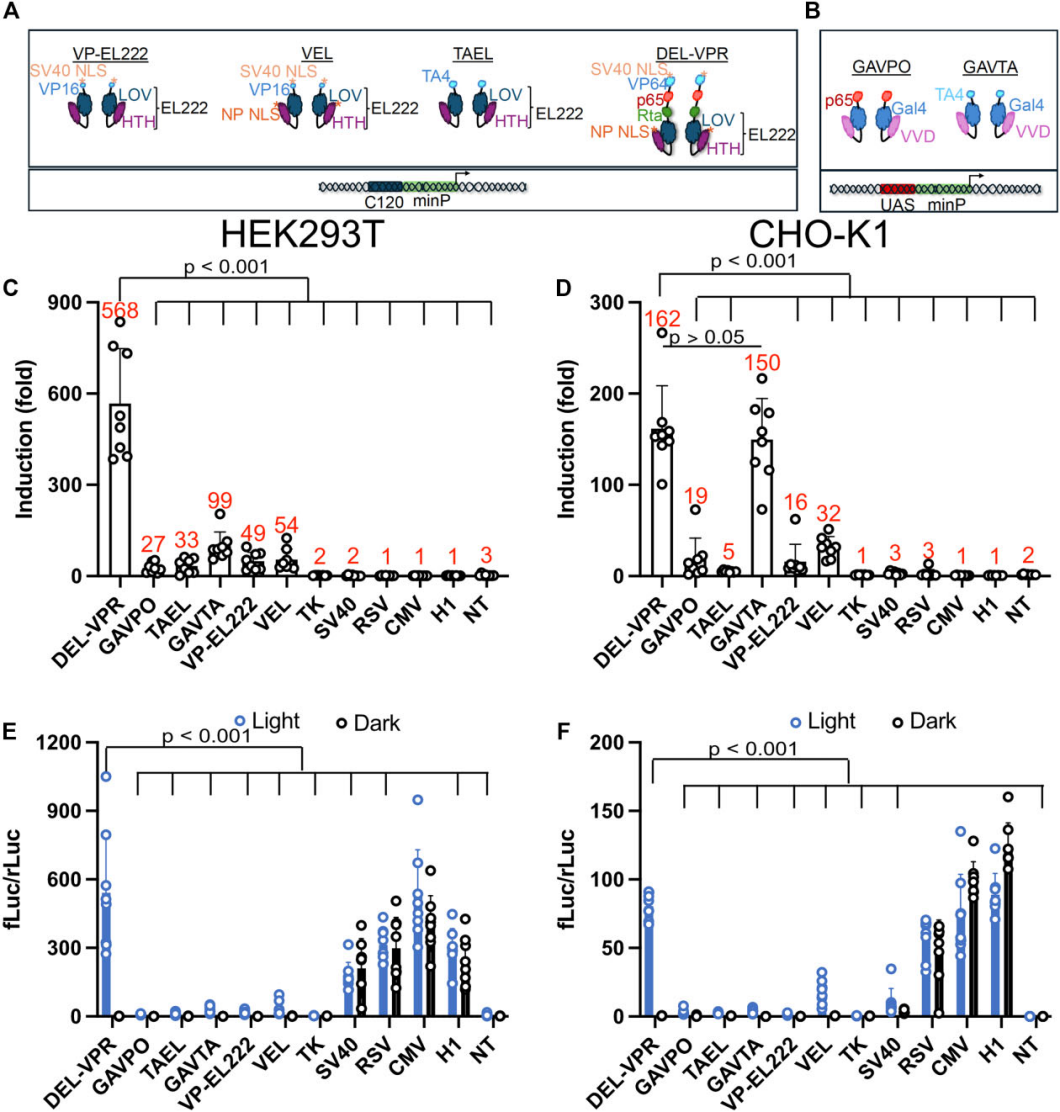
Comparison of various LOV-based photoswitches in HEK293T and CHO-K1 cells. (**A**) EL222-based photoswitches consist of a blue light-dependent LOV domain and an HTH DNA-binding domain, mediating gene expression by binding to the C120 sequence, which is located upstream of a minimal promoter (minP). The photoswitch VP-EL222 consists of an N-terminal SV40 NLS followed by VP16 and EL222. VEL consists of an N-terminal SV40 NLS, VP16, nucleoplasmin (NP), NLS, and EL222. TAEL consists of the transactivation domain TA4 and EL222. DEL-VPR comprises an SV40 NLS, the three transactivation domains VP64, p65, and Rta, as well as EL222 and a C-terminal NP NLS. (**B**) VVD-based photoswitches consist of the DNA-binding domain Gal4 and the blue light-dependent VVD domain. They mediate gene expression by binding to the UAS sequence, which is upstream of a minP. GAVPO consists of p65 fused to Gal4 and VVD. GAVTA contains TA4, Gal4, and VVD. Induction of light-mediated luciferase activity in panel (**C**) HEK293T and (**D**) CHO-K1 cells. The values indicate the induction of luciferase activity compared to the dark activity of various photoswitches and constitutive promoters. The cells were excited with continuous blue light (470 nm) for 9 h at 1500 μW/cm^2^. The data represent the ratio of light versus dark from fLuc normalized to SV40-driven renilla luciferase (rFluc) expression. Relative luciferase activity of firefly versus renilla luciferase of various experiments shown in panels (C, D) in HEK293T (**E**) and CHO-K1 cells (**F**). (C–F) Mean ± SD (*n* = 8). Dots represent means of triplicates from individual experiments. Statistical analysis was performed using one-way ANOVA and the Holm-Sidak post-hoc test. Indicated *P*-values represent comparison to DEL-VPR. (C, D) Red-written numbers show means of induction.

To optimize inducible gene expression for protein production, we developed an optogenetic tool for precise temporal control that achieves high expression levels while promoting protein functionality. We identified the bacterial LOV-based expression systems as promising candidates due to their compact size, natural occurrence of the chromophore, and proven performance. We investigated LOV-based photoswitches derived from EL222, fused to the transactivation domain VP16 with either a single SV40 NLS (VP-EL222) or a modified version incorporating an additional nucleoplasmin NLS (VEL; Fig. 1A; [29]). Additionally, we examined TAEL, a fusion of EL222 with a triplicated VP16 core (TA4; [46]). These tools were primarily optimized for the application in zebrafish. To enhance activation potential, we replaced VP16 with VPR (VP64-p65-Rta), known for its superior transcriptional activity compared to its constituent components [38, 46–49]. We then compared the EL222-based photoswitches with GAVPO (p65-Gal4-VVD; Fig. 1B) and GAVTA (TA4-Gal4-VVD), which are based on the VVD LOV receptor and have also been used in zebrafish [46]. Performance was assessed in a luciferase assay and benchmarked against common constitutive promoters.

fLuc activity, induced by photoswitches or constitutive promoters, was measured and normalized to SV40-driven, constitutively expressed renilla luciferase (rLuc) to account for transfection efficiency and cell number. DEL-VPR achieved a 570-fold induction in HEK293T and 160-fold in CHO-K1 under light versus dark conditions (Fig. 1C and D). For reference, in HEK293T cells, the other photoswitches induced luciferase activity by 30–100-fold upon light stimulation. In CHO-K1, GAVTA induced a 150-fold increase, while the other optogenetic tools led to inductions of merely 5–30-fold. As expected, the constitutive promoters and nontransfected controls showed no light dependency.

When fully induced by blue light, DEL-VPR reached levels comparable to or even exceeding common constitutive promoters (Fig. 1E and F). DEL-VPR significantly outperformed all other photoswitches in HEK293T and CHO-K1 cells, with no significant differences in dark activity among photoswitches in HEK293T were detected (Supplementary Fig. S1A). In CHO-K1, DEL-VPR dark activity was comparable to GAVPO, TAEL, and VEL but higher compared to GAVTA and VP-EL222 (Supplementary Fig. S1B). These data point to the especially low dark activity rather than the strength of induced expression as the main driver for GAVTA-mediated induction in CHO-K1.

These results demonstrate that DEL-VPR is a highly effective optogenetic tool for light-induced gene expression, achieving expression levels comparable to strong constitutive promoters such as CMV and H1 while maintaining low dark activity, with variations depending on the cell type.

### DEL-VPR drives light-inducible protein expression in iPSC-derived cells via AAV transduction

**Figure 2.**
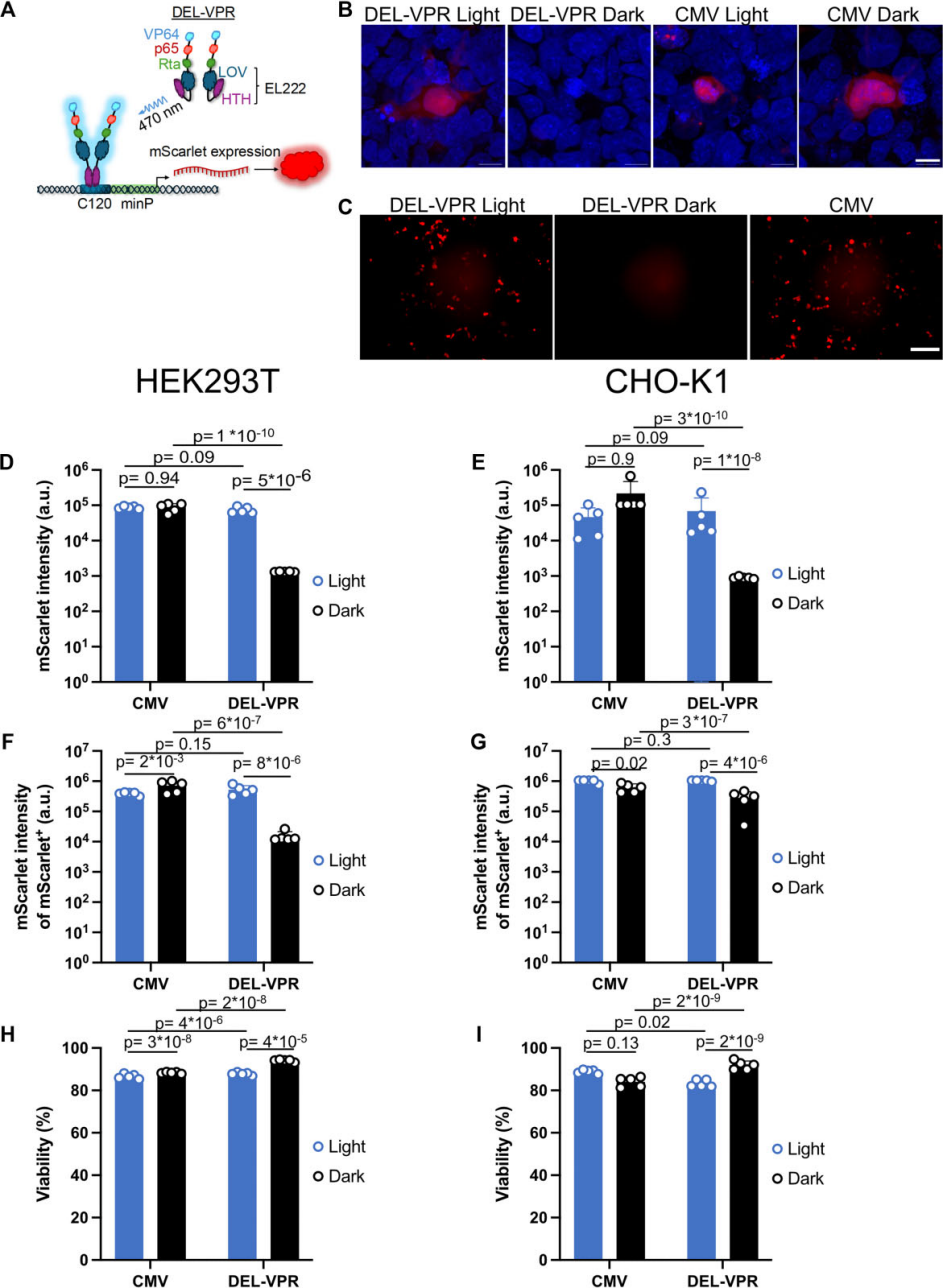
Single-cell analysis of DEL-VPR-mediated induction. (**A**) DEL-VPR is activated by blue light excitation (*λ* = 470 nm), and in turn, dimerizes and binds to the C120 sequence to induce mScarlet3 expression. (**B**) hiPSC-derived smNPCs transduced with AAV2/1-mScarlet3, either controlled via the light-inducible DEL-VPR or the constitutive CMV promoter, were exposed to blue light for 24 h before fixation, staining, and imaging. Light-exposed smNPCs transduced with the C120/DEL-VPR construct display robust mScarlet3 expression (red), as opposed to the dark control. CMV-driven constructs display expression independent of light exposure. Nuclei were stained with DAPI (blue). Scale bar: 10 μm. (**C**) Fluorescence images of HEK293T cells transfected with DEL-VPR and C120-mScarlet3 (encoded on the same plasmid) or CMV-mScarlet3, exposed to 24 h of blue light at 1500 μW/cm^2^ (always ON) or kept in the dark. Scale bar: 100 μm. mScarlet3 intensity, measured by flow cytometry, in panels (**D**) HEK293T or (**E**) CHO-K1 cells expressing DEL-VPR- or CMV-mediated mScarlet3 plus respective dark controls. Cells were continuously excited with blue light 24 h after transfection for 24 h at 1500 μW/cm^2^. mScarlet3 intensity of mScarlet3^+^ cells in (**F**) HEK293T or (**G**) CHO-K1 cells from experiments presented in panels (D) and (E). Viability of light-exposed (**H**) HEK293T or (**I**) CHO-K1 from experiments presented in panels (D) and (E) using Zombie Violet staining. (D–I) Mean ± SD (*n* = 5). Dots represent means of triplicates from individual experiments. Statistical analysis was performed using two-way ANOVA and the Holm-Sidak post-hoc test.

Building on our initial success using DEL-VPR to induce protein expression in immortalized cell lines, we next sought to explore whether this optogenetic system could be delivered via AAV, a critical step for application in cell types that require viral vectors for genetic manipulation. To this end, we engineered an AAV expressing either DEL-VPR together with the light-responsive reporter C120-mScarlet3 or a constitutively active CMV-mScarlet3 construct as a control (Fig. 2A). Upon transduction of hiPSC-derived smNPCs, red fluorescence was observed in cells expressing DEL-VPR and C120-mScarlet3 following blue light stimulation, while no signal was detected in the absence of light (Fig. 2B). In contrast, CMV-mScarlet3 expression was consistently visible regardless of light exposure. These results demonstrate that DEL-VPR can be efficiently packaged into AAV and functionally expressed in cell types that depend on viral transduction, paving the way for its application in more physiologically relevant systems such as primary cells and organoids.

### Single-cell analysis of DEL-VPR-mediated fluorescent reporter expression

The luciferase activity in Fig. 1 reflects cumulative expression from all cells in the well but lacks insights into cell-to-cell variability in expression levels and distribution. Thus, we conducted single-cell analysis using imaging and flow cytometry by measuring the fluorescence intensity of mScarlet3, driven by DEL-VPR. Of note, DEL-VPR and C120-mScarlet3 were encoded on the same plasmid to minimize variability from co-transfection.

In HEK293T and CHO-K1, a strong induction of mScarlet3 upon light stimulation was observed, visualized by fluorescence imaging (Fig. 2C, Supplementary Fig. S2). The transfection efficiency did not vary between experiments and reached 81% in HEK293T cells and 62% in CHO-K1 cells for CMV-mScarlet3, and 76% in HEK293T and 63% in CHO-K1 for DEL-VPR (Supplementary Fig. S3). Without light stimulation, mScarlet3-positive cells (mScarlet3^+^) were barely observed for DEL-VPR, whereas after illumination, they reached the level of CMV-driven mScarlet3^+^ cells.

Analysis of mScarlet3 expression using flow cytometry revealed that blue light exposure promoted mScarlet3 fluorescence intensities in DEL-VPR-transfected cells that were comparable to CMV-driven expression in both the total and mScarlet3^+^ populations (Fig. 2D–G). In contrast, cells kept in the dark exhibited low mScarlet3 intensities. Notably, in DEL-VPR-transfected cells, even the mScarlet3^+^ population showed significantly lower fluorescence in the dark compared to light-exposed cells (Fig. 2F–G). These findings were further supported by live-cell imaging (Supplementary Fig. S4). Altogether, these findings confirm that DEL-VPR has low basal activity, both in terms of the proportion of affected cells as well as expression intensity and promotes a strong expression upon light stimulation.

### Single-cell characterization of cyto- and phototoxicity

Photoactivation, transactivation domains, and blue light exposure can all contribute to cytotoxicity. To assess this, we performed a viability assay using flow cytometry on HEK293T and CHO-K1 cells transfected with DEL-VPR and C120-mScarlet3 (on a single plasmid; Fig. 2A) or CMV-mScarlet3 and constantly stimulated for 24 h with blue light (1500 μW/cm²) or kept in the dark.

DEL-VPR cells kept in the dark showed a higher viability than CMV-transfected cells and light-exposed DEL-VPR cells in both cell types, indicating that DEL-VPR itself does not exhibit cytotoxicity (Fig. 2H and I). In HEK293T cells, blue-light exposure mildly reduced cell viability, independently of the presence of DEL-VPR, whereas in CHO-K1 cells, only DEL-VPR-transfected cells showed reduced viability in the light compared to the dark condition. Taken together, we observed only a minimal cyto- and phototoxicity of DEL-VPR.

### Optimization of the light dose for maximal expression with minimal light exposure

**Figure 3.**
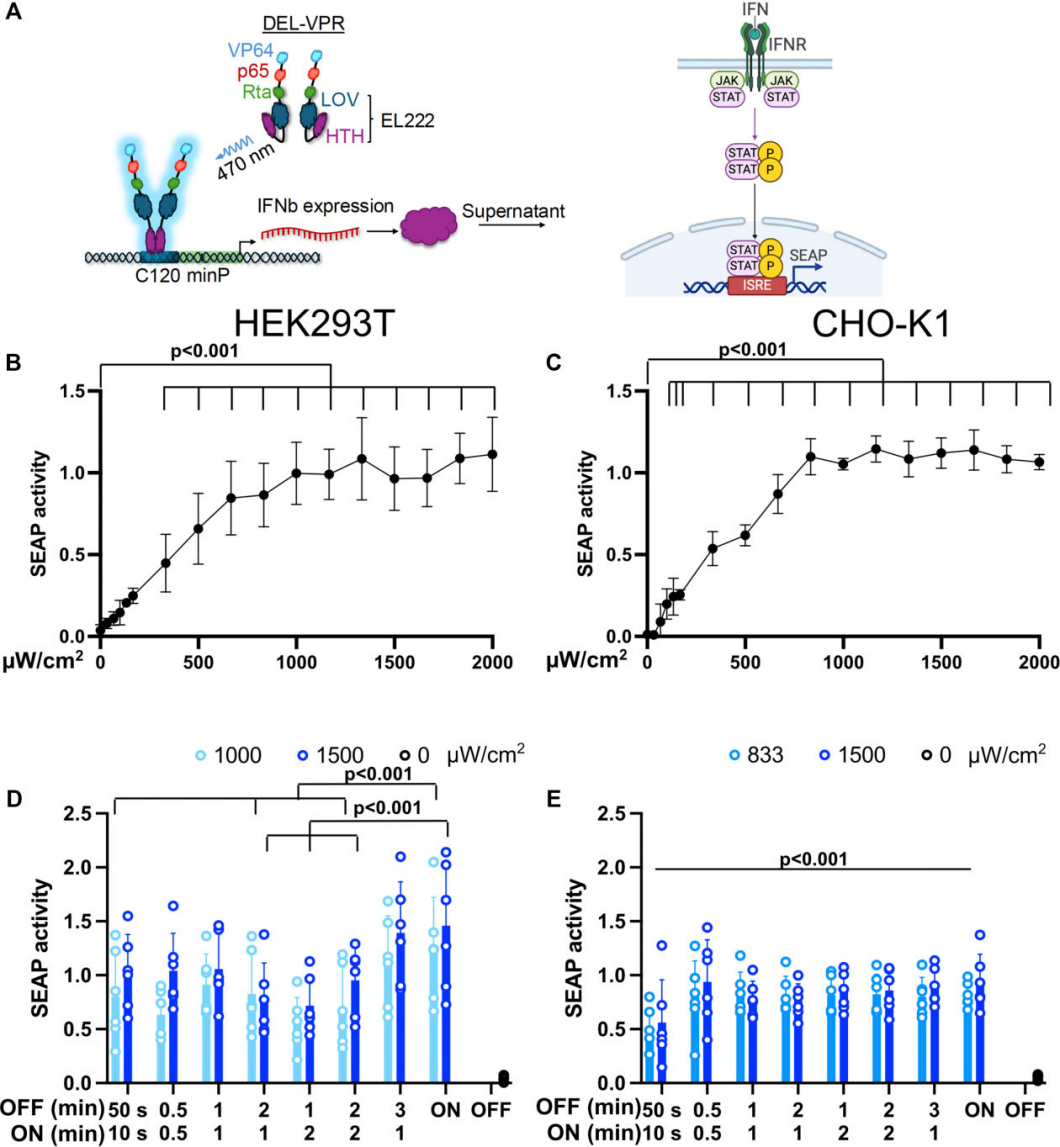
DEL-VPR mediates light-dose-dependent expression of functional recombinant proteins. (**A**) DEL-VPR is activated by blue light illumination (*λ*= 470 nm) and in turn, dimerizes and binds to the C120 sequence to induce interferon (IFN) expression. Functional IFN induces via an artificial IFN receptor (IFNR)/STAT signaling pathway the expression of SEAP, which can be detected in a colorimetric assay in high throughput. Dose-response curves of SEAP activity induced at indicated light intensities in panels (**B**) HEK293T and (**C**) CHO-K1 cells. Cells were excited 24 h after transfection for 24 h at indicated blue light intensities (always ON). The supernatant of these cells was added at a dilution of 1:100 on HEKblue IFN cells and incubated for 24 h. Values were normalized to CMV-driven SEAP activity of respective experiments. Mean ± SD (*n* = 6). Statistical analysis was performed using one-way ANOVA and the Holm-Sidak post-hoc test. Light-dependent SEAP activity induced at indicated light intensities and pulses in panels (**D**) HEK293T and (**E**) CHO-K1 cells. Cells were stimulated 24 h after transfection for 24 h at the indicated blue light intensities and pulses. The supernatant of these cells was added at a dilution of 1:100 on HEKblue IFN cells and incubated for 24 h. Values were normalized to CMV-driven SEAP activity of respective experiments. Mean ± SD (*n* = 6), dots show the mean of technical triplicates of individual experiments.

Although we did not observe substantial phototoxicity under the tested conditions, further minimizing cellular stress is an important goal for the potential use of DEL-VPR in long-term, large-scale bioproduction. To reduce the light exposure while maintaining the high inducibility of DEL-VPR, we investigated the lowest effective light dose. First, we conducted a dose-response experiment by varying light intensity under continuous exposure. To assess expression efficiency, we used light-induced expression of interferon beta (IFNβ), a protein relevant to bioproduction. In this assay, secreted IFNβ activates SEAP expression in reporter cells, with SEAP activity directly correlating to functionally active IFNβ levels (Fig. 3A).

SEAP expression was significantly induced at light intensities above 333 μW/cm² in HEK293T and above 100 μW/cm² in CHO-K1 (Fig. 3B and C, Supplementary Fig. S5). SEAP activity increased with higher light intensities, reaching saturation at 1000 μW/cm² in HEK293T and 833 μW/cm² in CHO-K1. At these intensities, SEAP levels matched those of the CMV condition, to which samples were normalized. Thus, blue light applied at 1000 μW/cm² is sufficient for maximal DEL-VPR induction.

After identifying these minimum light intensities for maximal DEL-VPR induction, we tested different light-pulse regimens to further reduce light exposure and minimize potential phototoxicity. These intensities were benchmarked against the previously used 1500 μW/cm² and normalized to CMV-driven expression levels in each experiment. As observed in continuous light conditions, no significant differences were detected between 1000 and 1500 μW/cm² in HEK293T or between 833 and 1500 μW/cm² in CHO-K1 under any pulsed-light regimen (Fig. 3D and E). In HEK293T, light pulses of 10 s/50 s, 1 min/1 min, 2 min/1 min, and 1 min/3 min (ON/OFF) at 1000 μW/cm² maintained IFNβ/SEAP expression comparable to continuous illumination (Fig. 3D). At 1500 μW/cm², pulses could be reduced to 10 s/50 s, 0.5 min/0.5 min, 1 min/1 min, 2 min/2 min, or 1 min/3 min (ON/OFF) without significant loss of expression. In CHO-K1, all pulse conditions except 10 s/50 s at 833 μW/cm^2^ preserved SEAP activity without significant reduction (Fig. 3E).

In summary, overall light exposure can be reduced by resorting to intermittent illumination with a 1 min/3 min ON/OFF duty cycle at 1000 μW/cm² for HEK293T and 833 μW/cm² for CHO-K1, thus decreasing total light dose by four-fold. This also allows reducing energy and thus heat input into the cell culture by optogenetic irradiation, which becomes particularly important in large-scale formats with low surface to volume ratios and, in turn, unfavorable efficiencies of heat dissipation caused by optogenetic control regimes.

### Optogenetic production of a monoclonal antibody

**Figure 4.**
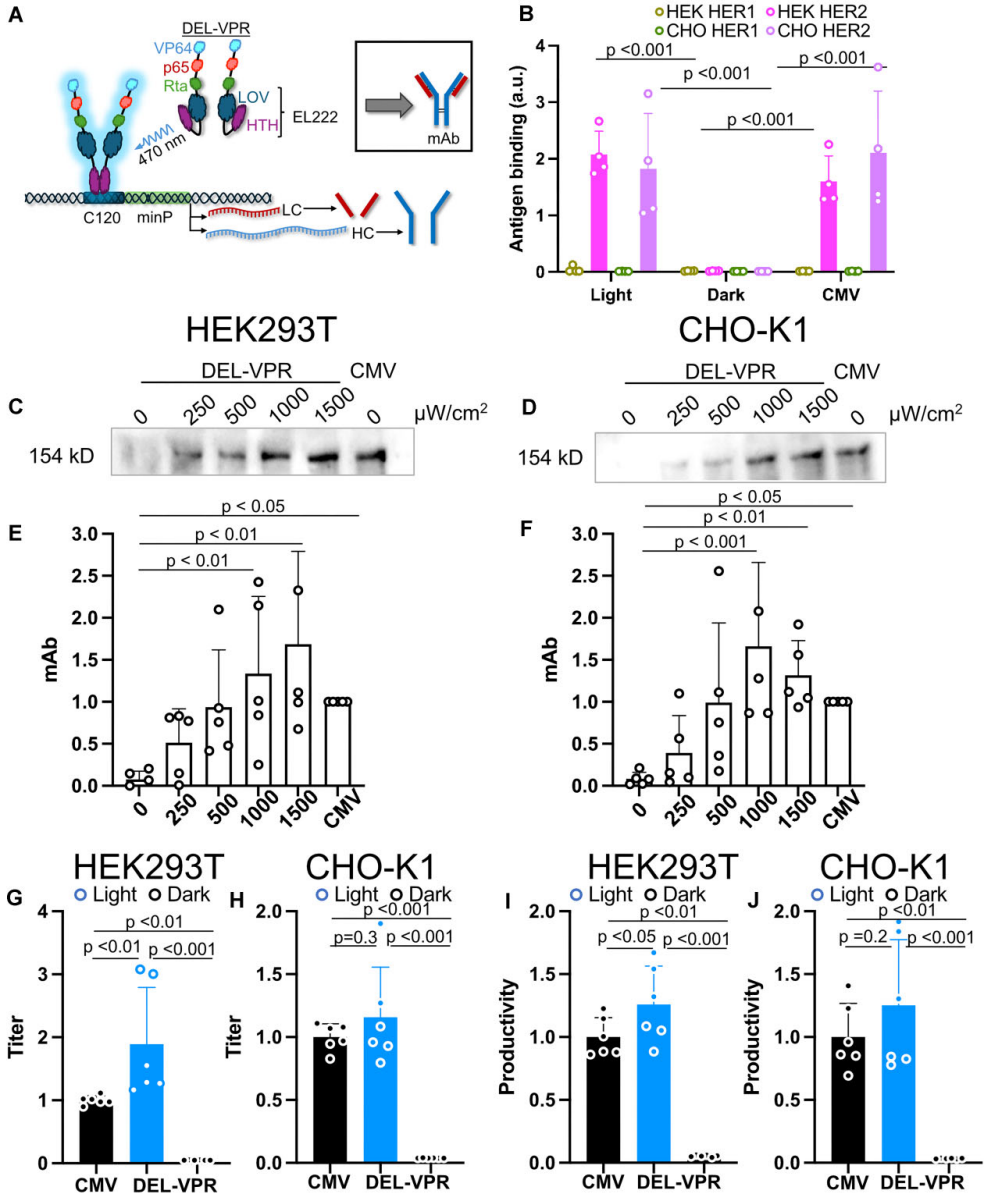
Light-dependent titration of mAb expression in HEK293T and CHO-K1. (**A**) DEL-VPR is activated by blue light excitation (*λ* = 470 nm), and in turn, dimerizes and binds to the C120 sequence. Subsequently, the LC and HC are expressed in a light dose-dependent manner, followed by their assembly into mAb in the ER. (**B**) Assessment of DEL-VPR and CMV-driven mAb functionality in HEK293T and CHO-K1 cells. The mAb antigen HER2 was coated on ELISA plates, and the bound mAb was detected. HER1 serves as a negative control. The absorbance of nontransfected cell supernatants was subtracted. The mAb, consisting of the light and heavy chains, was expressed in HEK293T (**C**) or CHO-K1 (**D**) either light-dependently under the control of the C120 promoter or constitutively under the control of the CMV promoter. The light-dependent conditions were excited at different intensities (μW/cm^2^) for 24 h (always ON). The samples were analyzed on a WB under nonreducing conditions using an anti-human IgG1 detection antibody coupled to HRP. The mAb bands of the WB for the HEK293T and CHO-K1 were quantified in panels (**E**) and (**F**), respectively. Panels (**G**) and (**H**) show the fold change of mAb titers of the light-induced (DEL-VPR light; 1500 μW/cm^2^), noninduced (DEL-VPR dark), or the constitutive (CMV) conditions after 24 h expression in HEK293T or CHO-K1, respectively, quantified by ELISA. The values were normalized to the CMV values. Panels (**I**) and (**J**) show the fold change of mAb productivity of the experiment presented in panels (G–H) in HEK293T and CHO-K1, respectively. The values were normalized to the CMV values. (B, E–J) Mean of $ \pm$ SD presented as bars, dots indicate individual values of each sample. Statistical analysis was performed using one-way ANOVA with the Holm-Sidak post-hoc test; (*n* = 4; B), (*n* = 5; E, F), or (*n* = 6; G–J).

As our data pinpointed DEL-VPR as a powerful tool for modulating gene expression in mammalian cells, we investigated whether more complex proteins, such as monoclonal antibodies (mAbs), widely used in biotherapeutics, could also be efficiently produced using this optogenetic strategy. We chose the human epidermal growth factor receptor 2 (HER2)-targeted mAb trastuzumab (IgG), also known as Herceptin, which is used to treat HER2-positive breast and gastric cancer by inhibiting the HER2 signaling pathway and thereby limiting proliferation [50, 51]. The mAb comprises two LCs and two HCs that assemble into a heterotetramer. We cloned the LC and HC downstream of the light-regulated promoter C120 and co-expressed the two chains under the control of DEL-VPR (Fig. 4A).

To confirm the functionality of light-induced and CMV-driven mAb, we performed an ELISA using HER2-coated plates as the target antigen. Both DEL-VPR (light) and CMV-driven mAb in HEK293T and CHO-K1 showed clear absorbance signals for HER2, whereas DEL-VPR samples incubated in the dark showed no signal (Fig. 4B). Specificity was verified using HER1, a nontarget antigen, which displayed no signal above the non-transfected control (used as normalization).

After confirming the mAb functionality, the dose-dependent inducibility was examined. In both HEK293T and CHO-K1 cells, mAb expression was not detectable in the dark (Fig. 4C–F). However, blue-light exposure increased mAb expression, assembly, and secretion into the supernatant in a light dose-dependent manner. In HEK293T cells, mAb expression was maximally induced at 1500 μW/cm^2^ blue light, while in CHO-K1 cells, it was already reached at 1000 μW/cm^2^ blue light and slightly dropped again at 1500 μW/cm^2^. At the maximal blue-light intensity, the expression levels reached or exceeded those afforded by the constitutive CMV promoter.

ELISA against human IgG demonstrated a significant increase in antibody titer upon light induction by DEL-VPR in HEK293T and CHO-K1 cells, while in the dark, the titer barely exceeded the value of the negative control used for normalization (Fig. 4G and H, Supplementary Fig. S6A and B). Similar results were observed for the productivity measured in pg/cell/day (Fig. 4I and J, Supplementary Fig. S6C and D). For these experiments, the cells were kept in the dark for the first 24 h after transfection before switching to light stimulation for 24 h. In contrast, for the constitutive promoters (CMV-DEL-VPR, CMV-mAb), the protein production occurred for a total of 48 h. However, even despite the shorter production time, the mAb titer and productivity were significantly increased by DEL-VPR compared to the constitutive CMV-driven expression in HEK293T (Fig. 4G and I, Supplementary Fig. S6A–D). However, in CHO-K1 cells, the titer and productivity were not significantly different between the DEL-VPR (light) and CMV (Fig. 4H and J, Supplementary Fig. S6B and D). Thus, our data demonstrate that DEL-VPR allows a tightly controlled, light-dependent induction of functional mAb expression, reaching or even surpassing the level of strong constitutive expression mediated by a CMV promoter.

### Optogenetic production of a bispecific antibody

Having successfully demonstrated the light-dependent expression of mAb, we next applied our approach to more complex biopharmaceuticals like a bispecific antibody (bsAb). Since one of the driving factors for the high production costs of bsAb is a contamination of unwanted by-products (e.g. HCscFv homodimers), we investigated whether we could modulate bsAb complex assembly by light-mediated induction. To establish this, we initially tested the light regulation of all bsAb chains simultaneously.

**Figure 5.**
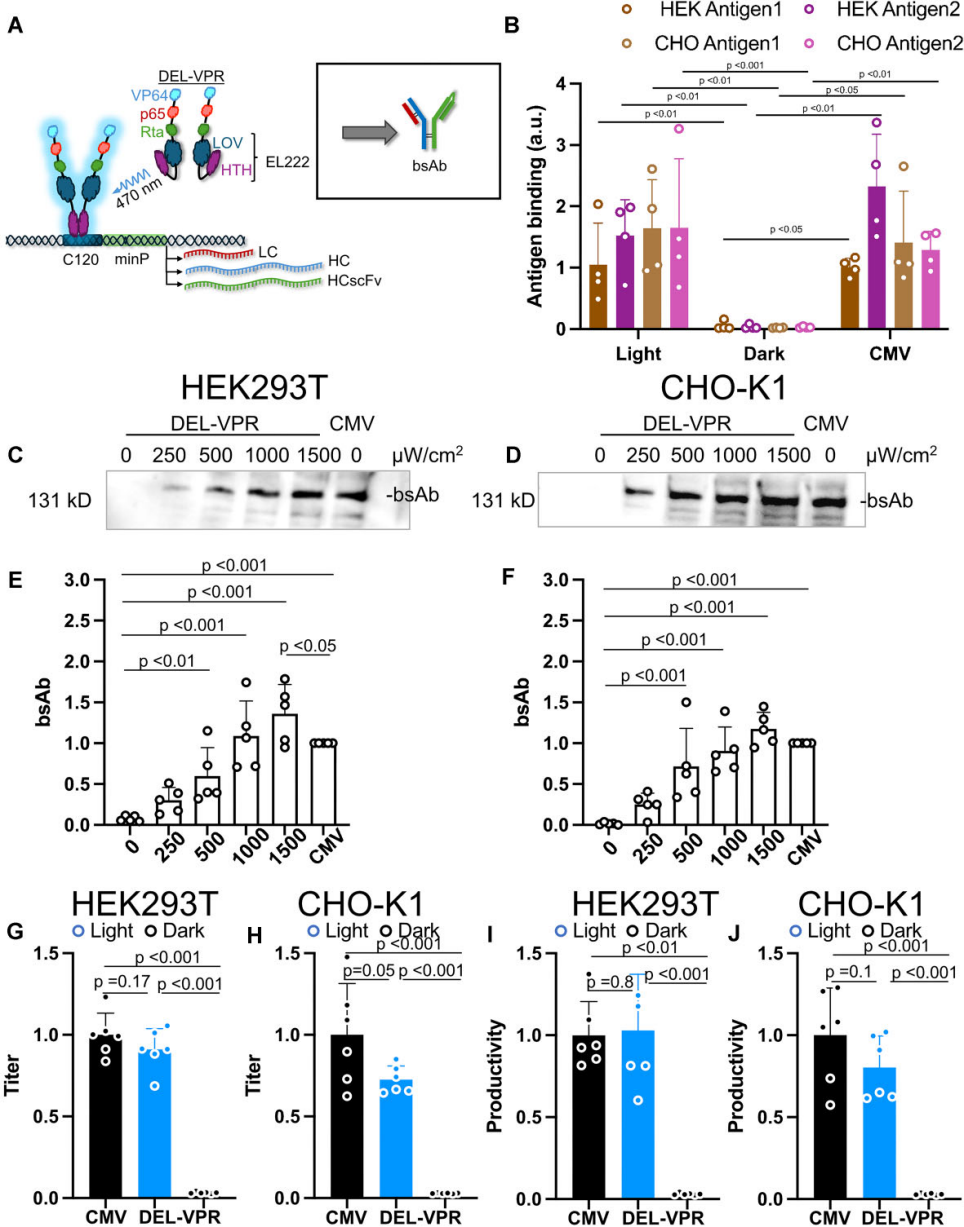
Light-dependent titration of bsAb expression in HEK293T and CHO-K1. (**A**) DEL-VPR is activated by blue light stimulation (*λ*= 470 nm), and in turn, dimerizes and binds to the C120 sequence. Subsequently, the LC, HC, and HCscFv are expressed in a light dose-dependent manner, followed by their assembly into bsAb in the ER. (**B**) Assessment of DEL-VPR and CMV-driven bsAb functionality in HEK293T and CHO-K1 cells. The two distinct antigens were separately coated on independent ELISA plates, and bound bsAb was detected. The absorbance was subtracted from that of the non-transfected cells’ supernatant. The bsAb was either expressed in HEK293T (**C**) or CHO-K1 (**D**), either light-dependently under the control of DEL-VPR or constitutively under the control of the CMV promoter. Cells were excited at different intensities (μW/cm^2^) for 24 h (always ON). The samples were analyzed on a WB under nonreducing conditions using an anti-human IgG1 detection antibody coupled to HRP. The bsAb bands of the WB for the HEK293T and CHO-K1 were quantified in panels (E) and (F), respectively. Panels (G) and (H) show the fold change of bsAb titer of the light-induced (DEL-VPR; 1500 μW/cm^2^), noninduced (DEL-VPR dark), or constitutive (CMV) conditions after 24 h expression in HEK293T or CHO-K1, respectively, quantified by ELISA. The values were normalized to the CMV samples. Panels (I) and (J) show the fold change of bsAb productivity of the experiment presented in panels (G) and (H) in HEK293T and CHO-K1, respectively. The values were normalized to the CMV samples. (E–J) Mean $ \pm$ SD presented as bars, dots indicate individual values of each sample. Statistical analysis was performed using one-way ANOVA with the Holm-Sidak post-hoc test, (*n* = 4, B), (*n* = 5, D–F), or (*n* = 6, G–J).

We chose a trimeric bsAb (IgG), comprising an LC and HC on one side and an HCscFv on the other side. We cloned the three constituent bsAb chains downstream of the light-responsive promoters C120 and co-expressed them with DEL-VPR (Fig. 5A).

To confirm the functionality of light-induced and CMV-driven bsAb, we performed an ELISA using coated plates with each of the antigens separately as the prey. Both DEL-VPR (light) and CMV-driven bsAb in HEK293T and CHO-K1 showed clear absorbance signals for both antigens, whereas DEL-VPR samples incubated in the dark showed no signal (Fig. 5B). This data demonstrates the bispecific functionality of the produced antibodies.

After verifying the functionality of the bsAb, we analyzed the light-regulated expression on protein level using WBs. In both HEK293T and CHO-K1 cells, bsAb expression driven by DEL-VPR and secretion were barely detectable in the dark but significantly increased in a light dose-dependent manner, as observed by trimeric bsAb complex assembly in the supernatant (Fig. 5C–F). For both HEK293T and CHO-K1 cells, maximal bsAb expression was observed at 1500 μW/cm². In HEK293T cells, the bsAb level was significantly higher in the DEL-VPR samples (1500 μW/cm²) compared to the CMV-bsAb control (Fig. 5C and E). The light-dependent bsAb induction by DEL-VPR also emerged in stably integrated HEK293T (Supplementary Fig. S7). In CHO-K1 cells, the DEL-VPR-induced samples showed a tendency towards higher expression than the CMV samples (Fig. 5D and F). Strikingly, DEL-VPR-mediated expression resulted in the predominant assembly of the trimeric bsAb in the supernatant.

DEL-VPR led to a significant induction of the bsAb titer under blue light compared to the dark by 30-fold and 34-fold while increasing the productivity by 32-fold and 33-fold in HEK293T and CHO-K1, respectively (Fig. 5G–J and Supplementary Fig. S8). The antibody titer was not significantly different between the DEL-VPR-induced and CMV-driven expression in HEK293T cells (Fig. 5G and Supplementary Fig. S8A), whereas in CHO-K1 cells, the bsAb levels were slightly higher in the CMV-driven expression compared to the DEL-VPR-driven expression (Fig. 5H and Supplementary Fig. S8B). The productivity in pg/cell/day did not differ between the constitutive and the DEL-VPR-induced expression (Fig. 5I and Supplementary Fig. S8C and D). As described above, the constitutive expression occurred over 48 h, whereas the DEL-VPR-induced expression by light occurred over 24 h.

In summary, these data demonstrate that DEL-VPR can be used as an optogenetic tool for the tunable expression of large functional protein complexes, reaching levels of constitutive protein expression driven by, e.g. a CMV promoter. By allowing to switch on bsAb expression during a particular desired time window, DEL-VPR stands to reduce the metabolic burden that can occur with a static high expression using constitutive promoters such as CMV.

### bsAb complex composition can be modulated by light

**Figure 6.**
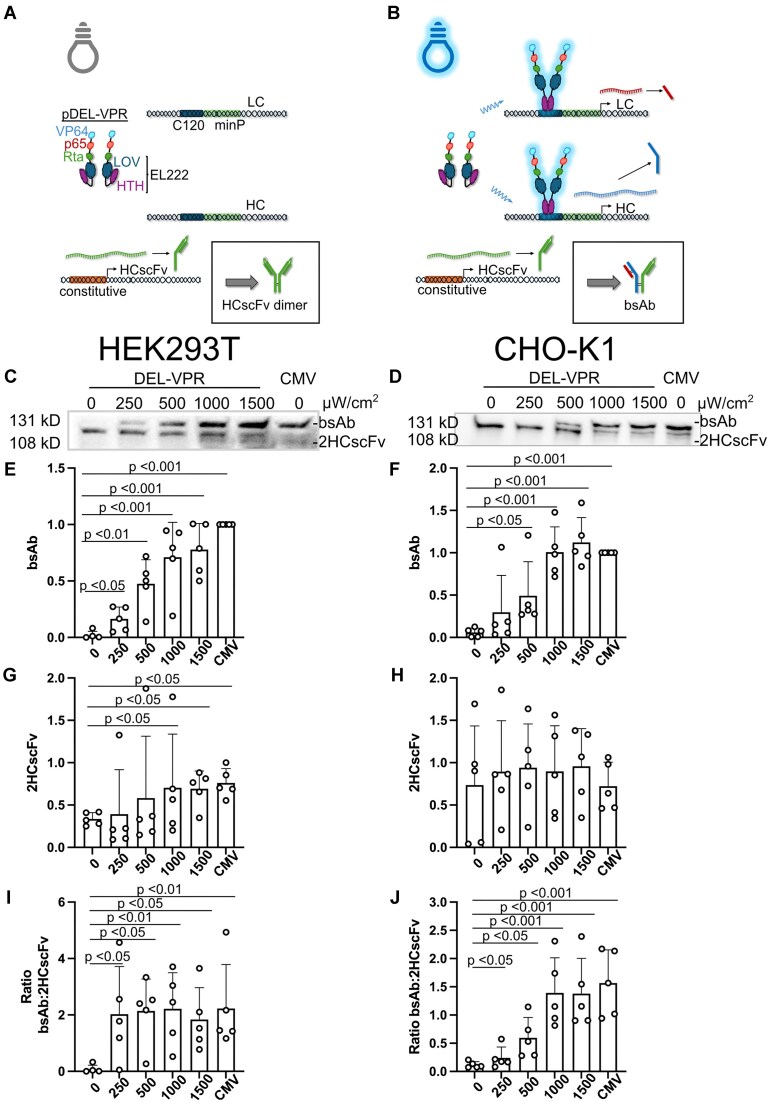
Light-dependent titration modulation of bsAb complexes in HEK293T and CHO-K1. (**A**) In the dark, the DEL-VPR stays inactive. Therefore, the light-dependent expression of the LC and HC is not induced, while the HCscFv is constitutively expressed. Consequently, without the LC and HC, the HCscFv is assembled in dimers. (**B**) DEL-VPR is activated by blue light stimulation (*λ*= 470 nm), dimerizes, and binds to the C120 sequence. Subsequently, the LC and HC are expressed in a light intensity-dependent manner. Additionally, the HCscFv is expressed constitutively. Consequently, in the light, the trimeric bsAb is assembled. The bsAb was expressed in HEK293T (**C**) or CHO-K1 (**D**) either partially light-dependently (light-dependent LC/HC + constitutive HCscFv) under the control of DEL-VPR or all chains constitutively under the control of the CMV promoter. The light-dependent conditions were excited at different intensities (μW/cm^2^) for 24 h (always ON). The different bsAb complexes were analyzed on a WB under nonreducing conditions using an anti-human IgG1 detection antibody coupled to HRP. The bsAb bands of the WB for the HEK293T and CHO-K1 were quantified in panels (**E**) and (**F**), respectively. The bands of HCscFv dimers for the HEK293T and CHO-K1 were quantified in panels (**G**) and (**H**), respectively. The ratio of bsAb:HCscFv dimers for the HEK293T and CHO-K1 were quantified in panels (I) and (J), respectively. (E–J) Mean $ \pm$ SD (*n* = 5); dots indicate the individual values of each sample. Statistical analysis was performed using ANOVA with the Holm-Sidak post-hoc test.

When we induced all three bsAb chains at the same time (Fig. 5B–E), we observed the trimeric bsAb as the predominant product. In addition to regulating the production of the bsAb, we examined whether the composition of the bsAb complexes could be modified by light, since by-products represent a major factor impeding purification and yield in bsAb production. To this end, we constitutively expressed the HCscFv chain combined with the light-dependent expression of LC/HC driven by DEL-VPR (Fig. 6A and B). This enables a light-dependent transition from the HCscFv dimer in the dark to the trimeric bsAb complex in the presence of light, thereby establishing the proof of concept for light-dependent modulation of protein complex assembly.

In the dark, only the constitutively expressed HCscFv was produced and subsequently assembled and secreted as HCscFv dimers into the supernatant in both HEK293T and CHO-K1 cells (Fig. 6C–F). We observed HCscFv dimers in all DEL-VPR conditions in the dark (Fig. 6C and D), but the expression of the LC and HC in a light dose-dependent manner resulted in a switch from the HCscFv dimers to the trimeric bsAb. This was also demonstrated by the ratio of bsAb to HCscFv dimer, which increased in a light dose-dependent manner (Fig. 6C, D, G, and H). At maximal expression (1500 μW/cm^2^), both the bsAb levels and the ratio of bsAb to HCscFv dimers reached levels like those for CMV-driven expression (Fig. 6C–F, I, and J).

In conclusion, our data show that DEL-VPR allows not only dynamic regulation of the amount of biopharmaceuticals produced but also their correct assembly into protein complexes. The use of DEL-VPR, therefore, offers the possibility to dynamically modify the composition of bsAb.

## Discussion

### Regulation of gene expression benefits bioproduction

Over the past decade, biopharmaceuticals have become increasingly important for treating life-threatening conditions such as cancer and autoimmune diseases. However, their widespread use remains constrained by high production costs. To overcome this challenge, extensive efforts have focused on increasing biopharmaceutical titers and lowering manufacturing expenses. One effective strategy has been to restrict recombinant gene expression, such as mAbs or other complex proteins, to the production phase alone. This approach minimizes the metabolic burden during the earlier selection and expansion stages, thereby improving the identification and viability of high-producing clones [5, 52, 53]. Another innovation involves a “gas and brake pedal” model to regulate cell growth, which has been shown to further enhance recombinant protein yields [54].

These advancements have in common that they primarily rely on chemically inducible gene expression systems. Chemically induced gene expression, e.g. the frequently used tetracycline/doxycycline system, is highly optimized [7] and provides strong induction of up to ∼100-fold [6, 55] while having a low basal activity in the absence of their inducer [56]. Irrespective of these benefits, chemical inducers face significant limitations that complicate application and scalability in many industrial settings. Not least, the scope for automation of induction is limited, the costs of GMP-grade inducers are high, and inducer removal is cumbersome as it often necessitates replacing the entire medium, which incurs substantial costs and slow deactivation kinetics. Against this backdrop, non-chemical induction methods have been explored (for review, see [11]).

Optogenetic systems provide high temporal resolution of induction and, thereby, enable rapid and reversible ON/OFF cycles, which are difficult to achieve with chemical methods. This dynamic control is particularly beneficial for expressing complex biopharmaceuticals, where post-translational modification and protein folding are often the limiting steps (57). Temporally precise regulation can minimize the accumulation of misfolded or toxic protein aggregates, thereby improving both yield and product quality. Moreover, optogenetic control is well-suited for high-throughput screening in drug discovery, where tight, temporal, and quantitatively tunable expression of target genes is essential.

### Optogenetic tools to control gene expression

Notwithstanding significant progress [11], optogenetic expression platforms still require optimization, particularly in terms of robustness and induction strength. We initially opted for the blue-light-induced EL222 transcription factor as it uses widely recurring flavin nucleotides as chromophores and does not target endogenous DNA but rather binds to the artificial promoter C120, thereby affording consistent binding properties [29, 34, 35, 58]. VP-EL222 (VP16 fused to EL222) and its modified version VEL (VP16-EL222 with an additional NLS) have previously achieved 100-fold induction with low basal activity in the dark [29, 34]. So far, neither absolute values nor comparisons with constitutive promoters have been published; however, the relative changes we observe for those tools after induction of gene expression by light are largely consistent with the literature. Using luciferase activity in mammalian cells as a read-out for gene expression, we determined a 50-fold increase with VP-EL222. However, when using a fluorescent reporter as a readout, the induced expression was below the detection limit, in line with Pulgarin *et al.*, who reported that VP-EL222 failed to induce mCherry expression in HEK293 cells [38]. Although other studies demonstrated VP-EL222-driven expression of fluorescence reporters in zebrafish and yeast [29, 38, 46], deploying the VP-EL222 system in mammalian cells might be difficult.

To address this shortcoming, we devised the DEL-VPR system by replacing the VP16 activation domain with the VPR tandem, which previously mediated high induction levels in various contexts [40, 41, 43]. Light-stimulated DEL-VPR drove 570- and 160-fold increases in luciferase activity in HEK293T and CHO-K1, respectively, and enabled robust and highly titratable induction of fluorescent proteins as well as monoclonal and bispecific antibodies. To our knowledge, this is the first report of an optogenetic system reaching or surpassing CMV-driven expression levels. At the same time, DEL-VPR retains the low basal activity of the preceding VP-EL222 and VEL setups. High expression strength is thus maintained without compromising light-dependent regulation. Consequently, DEL-VPR combines optimal properties in terms of strength, kinetics, and tightness, making it an ideal candidate for modulating large-scale bioproduction.

Beyond its strong potential for regulating bioproduction, DEL-VPR also serves as a highly versatile tool for research applications. Unlike many other optogenetic systems, it is a single-component system, which is small enough for packaging into viral vectors, as we demonstrated here using AAV (Fig. 2B). This allows not only the expression in immortalized cell lines but also in more challenging models, including primary cells, organoids, and even *in vivo* systems, which proved to be useful in a recent approach in T cells to perform CRISPR/Cas-base editing [38]. Furthermore, the low cell-to-cell variability and minimal photo- and cytotoxicity of the system further contribute to the favorable properties of DEL-VPR for studying protein function as well as signaling pathways and cellular regulations. Based on these properties, DEL-VPR could be particularly useful to express cytotoxic gene products.

Concurrent approaches for the optogenetic control of mammalian gene expression frequently rely on pairs of proteins that enter interactions once prompted by light. The red-light-responsive plant phytochromes and blue-light-responsive plant cryptochrome 2, together with their respective partner proteins, have been particularly widely deployed in this capacity [59, 60]. Notwithstanding differences in their implementation, pertinent strategies generally connect one of the interacting partners to a DNA-binding domain and the other to a trans-activating domain. Light promotes the assembly of the two components at target promoters and, thereby, ushers in transcription. Though powerful in principle, a two-component system depends on the intracellular expression, concentration, and localization of the light-responsive system components. Temporal and spatial imbalances, e.g. resulting from varying transfection efficiency and cell-to-cell heterogeneity, stand to strongly affect the system response. By contrast, EL222-based approaches, such as DEL-VPR advanced at present, employ a single, compact polypeptide component, thereby greatly reducing these challenges.

## Conclusions

In summary, DEL-VPR represents a powerful new tool for light-controlled gene expression with immediate applications in bioproduction and research. Although optogenetic regulation is still in its infancy and not yet fully integrated into industrial bioprocesses, its modularity, tunability, and low background activity make it a promising candidate for next-generation bioproduction platforms. We expect optogenetics to significantly impact and advance the development of future bioproduction systems. The growing implementation of in-process monitoring and control opens exciting opportunities for DEL-VPR to support dynamic, feedback-driven production workflows [61]. By coupling gene expression to real-time indicators of cellular stress, growth, viability, or protein complex composition, DEL-VPR could enable more responsive and efficient manufacturing processes, thus paving the way for higher product quality, reduced costs, and broader accessibility of biopharmaceuticals.

## Supplementary Material

gkaf546_Supplemental_File

## Data Availability

The original western blots and live-cell imaging picture, as well as the Excel files from the analysis, are available (https://doi.org/10.5281/zenodo.15237266).
