# Supplementary material for: Potent optogenetic regulation of gene expression in mammalian cells for bioproduction and basic research
Source: Nucleic Acids Res. 2025 Jun 30;53(12):gkaf546. doi: 10.1093/nar/gkaf546 (PMC12207406; doi:10.1093/nar/gkaf546)
Supplement: gkaf546_Supplemental_File [file gkaf546_supplemental_file.pdf]

Supplementary Information

Supplementary Table 1. Primer sequences.

Plasmid	Vector	Insert	Primer FW	Primer RV
5xC120-minP-mCherry	pcDNA3.1	mCherry	ggtggaattcgCCACCATGGTGAGCAAGGGC	cttTCTAGATTACTTGTACAGCTCGTCC
5xC120-minP-LC(mAb)	5xC120-minP-FLuc	LC (mAb)	ttttttGGCGCGCCTAATATTGCCACCATGTTGCCATCACAACTCATTGGGTTTCTGC	aaaaaaCGCCGGCGTACTAGTCTAACACTCTCCCCTGTTGAAGCTCTTTGTGACG
5xC120-minP-HC(mAb)	5xC120-minP-FLuc	HC (mAb)	ttttttGGCGCGCCTAATATTGCCACCATGGACTGGACCTGGAGGATCCTCTTCT	aaaaaaCGCCGGCGTACTAGTTCATTTACCCGGAGACAGGGAGAGGCT
CMVp-LC(mAb)	pcDNA3.1	LC (mAb)	ttttttCTCGAGACATTGATTATTGACTAGTTATTAATAGTAATCAATTACGGGGTC	aaaaaaGGCGCGCCGATCTGACGGTTCACTAAACGAGCTCTGC
CMVp-HC(mAb)	pcDNA3.1	HC (mAb)	ttttttCAATTGACATTGATTATTGACTAGTTATTAATAGTAATCAATTACGGGGTC	aaaaaaGGCGCGCCGATCTGACGGTTCACTAAACGAGCTCTGC

Supplementary Figures

Supplementary Figure 1

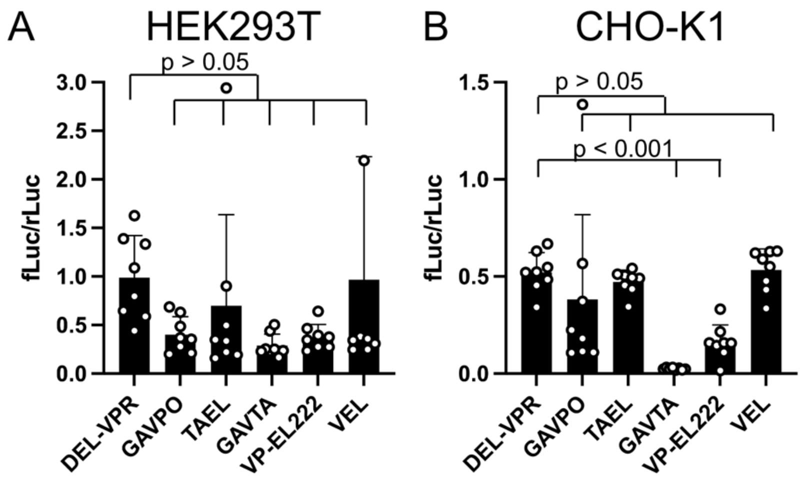

Supplementary Figure 1: Comparison of dark activity of various LOV-based photoswitches. Comparison of dark fLuc activity normalized to rLuc from various photoswitches in A) HEK293T and B) CHO-K1 cells. Bars represent the mean of six independent experiments (n = 8) ± SD. Dots represent means of triplicates from individual experiments. Statistical analysis was performed using one-way ANOVA and the Holm-Sidak post-hoc test. Indicated p-values represent comparison to DEL-VPR.

Supplementary Figure 2

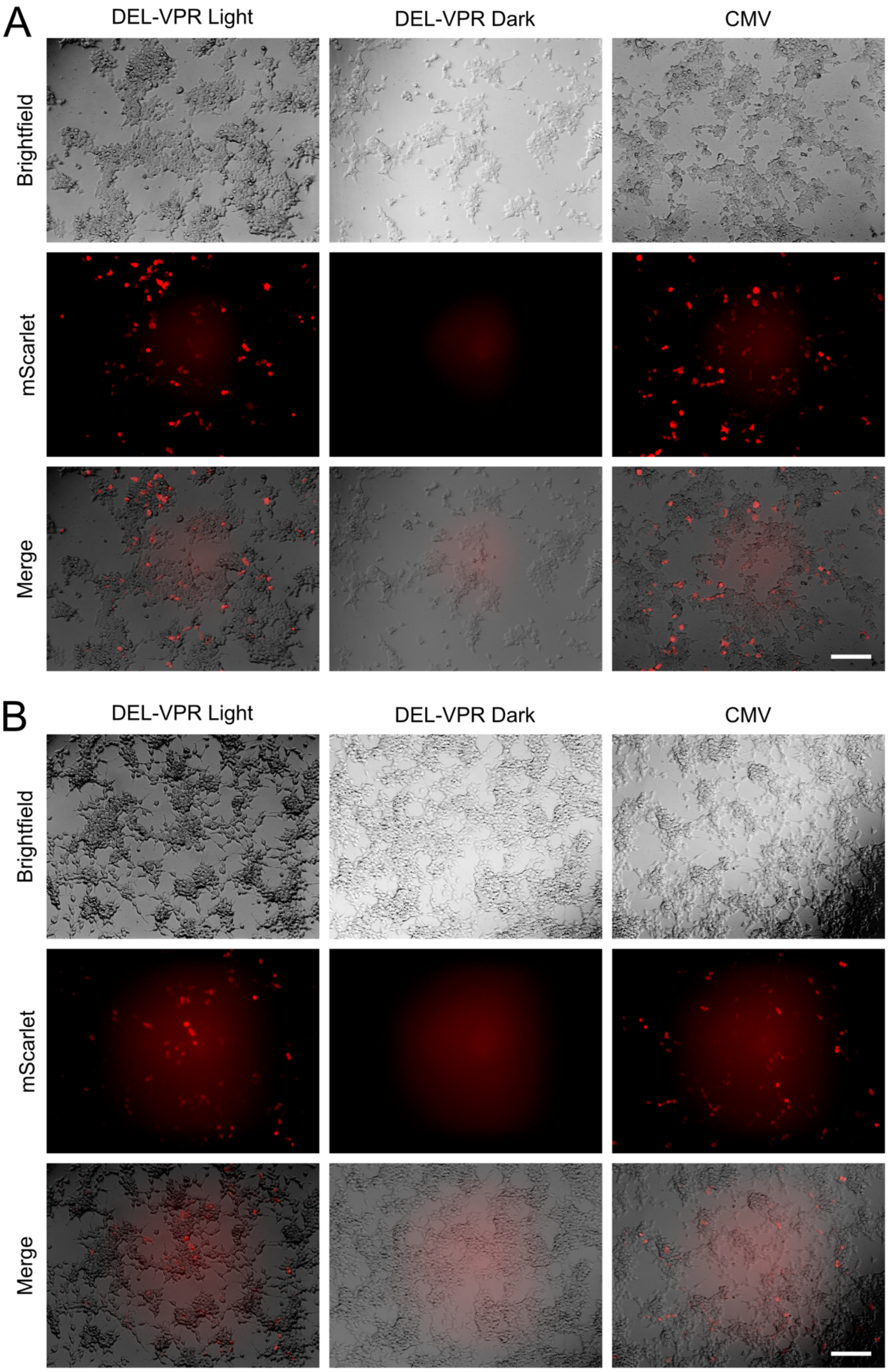

Supplementary Figure 2: **mScarlet3 induction in HEK293T and CHO-K1.** Brightfield and fluorescent (red, mScarlet) images of A) HEK293T and B) CHO-K1 expressing mScarlet3 either in a DEL-VPR mediated manner or CMV-driven in after 24 h of blue light exposure (1500 µW/cm2, always ON) or kept in the dark. Scale bar: 100 µm.

Supplementary Figure 3

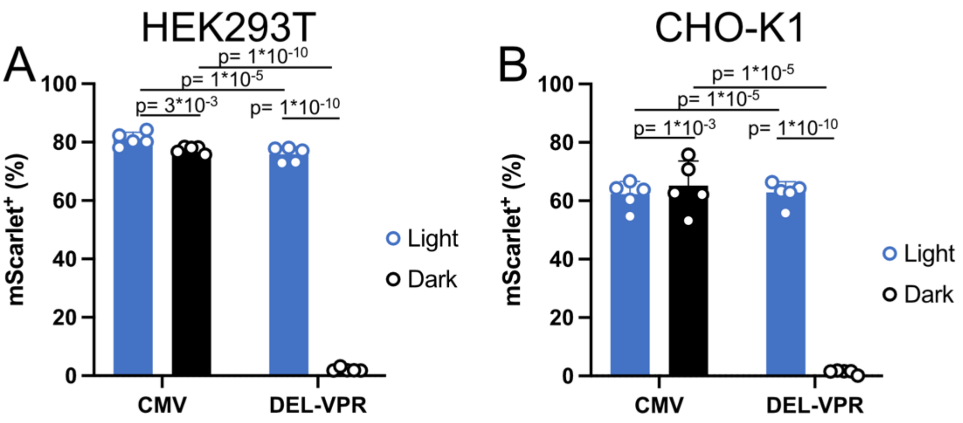

Supplementary Figure 3: Transfection efficiency of mScarlet3 expression in HEK293T and CHO-K1 cells. DEL-VPR was activated by blue light excitation (λ= 470 nm) and in turn, dimerizes and binds to the C120 sequence to induce mScarlet3 expression. Percentage of mScarlet3-positive (mScarlet3^{+}) A) HEK293T or B) CHO-K1 expressing DEL-VPR- or CMV- mediated mScarlet3 plus respective dark and non-transfected (NT) controls, measured by flow cytometry. Cells were continuously excited with blue light 24 h after transfection for 24 hours at 1500 μW/cm^2. Mean ± SD (n = 5). Dots represent means of triplicates from individual experiments. Statistical analysis was performed using two-way ANOVA and the Holm-Sidak post-hoc test.

Supplementary Figure 4

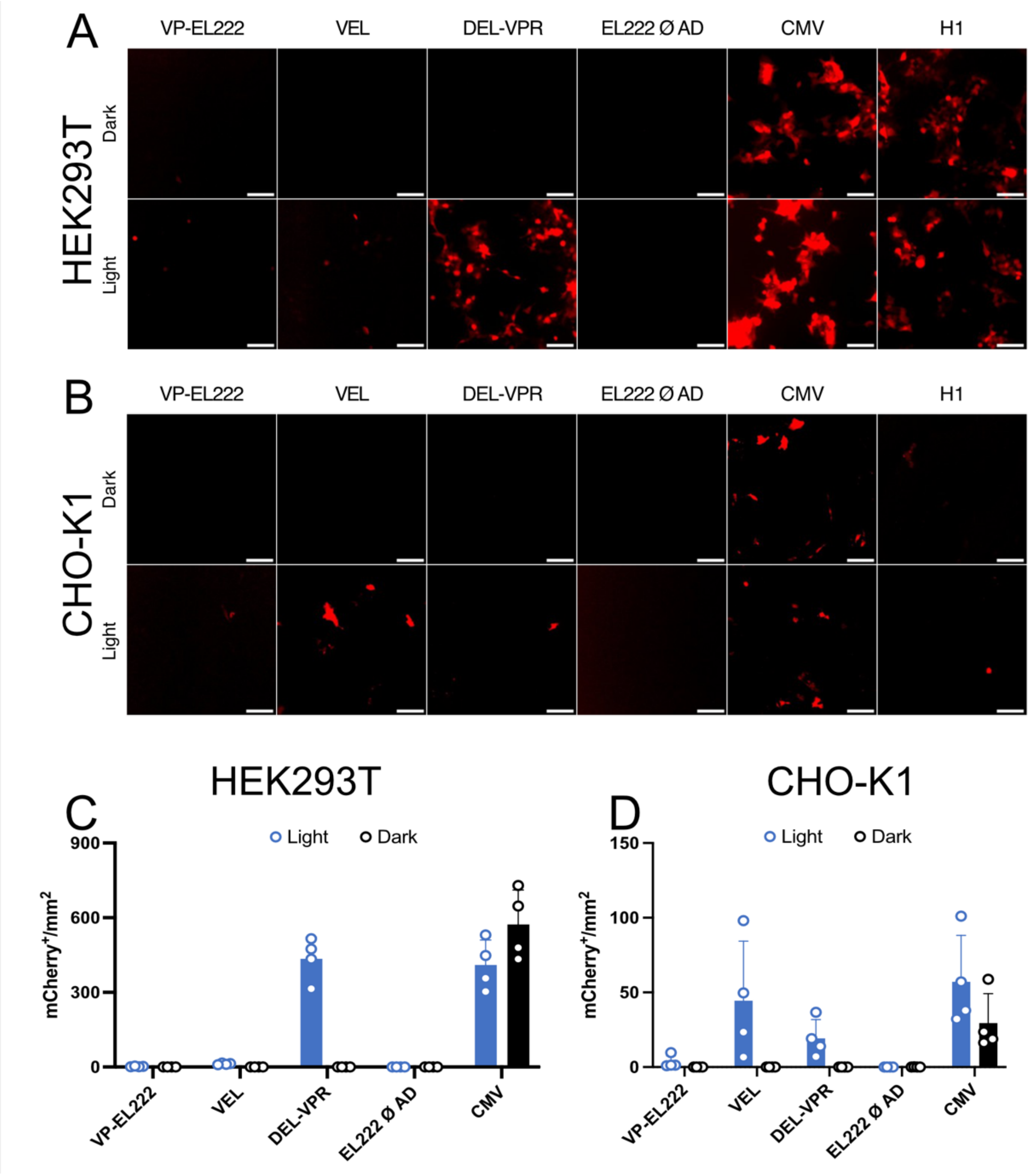

Supplementary Figure 4: Light-dependent fluorescent reporter expression in HEK293T and CHO-K1

Fluorescence levels were measured in HEK293T (A, C,) and CHO-K1 (B, D) cells, which transiently expressed mCherry either in a light-dependent fashion or under the control of different constitutive promoters. We compared different versions of the blue-sensitive photoswitch EL222—VP-EL222, VEL, DEL-VPR and EL222 without additional activation domain—and the strong and medium constitutive promoters CMV and H1, respectively. For the light condition, samples were excited for 32 h using blue light (λ= 470 nm) with a constant intensity (1500 µW/cm2), while for the dark condition, samples were kept in the dark for the entire time. Live cell image collection started 8 h after the beginning of the light excitation (+) and continued for a total of 48 h. For the last 24 h, all the samples were kept in the dark (-). In A) and B) are shown examples of the images collected and analyzed. Scale bar: 100 µm. C) and D) represent the mean values relative to the last time point (48 h) of the light and the dark conditions compared. Data are presented as lines with markers or as bar graphs, showing mean ± SD, and with dots indicating individual values of each sample.

Supplementary Figure 5

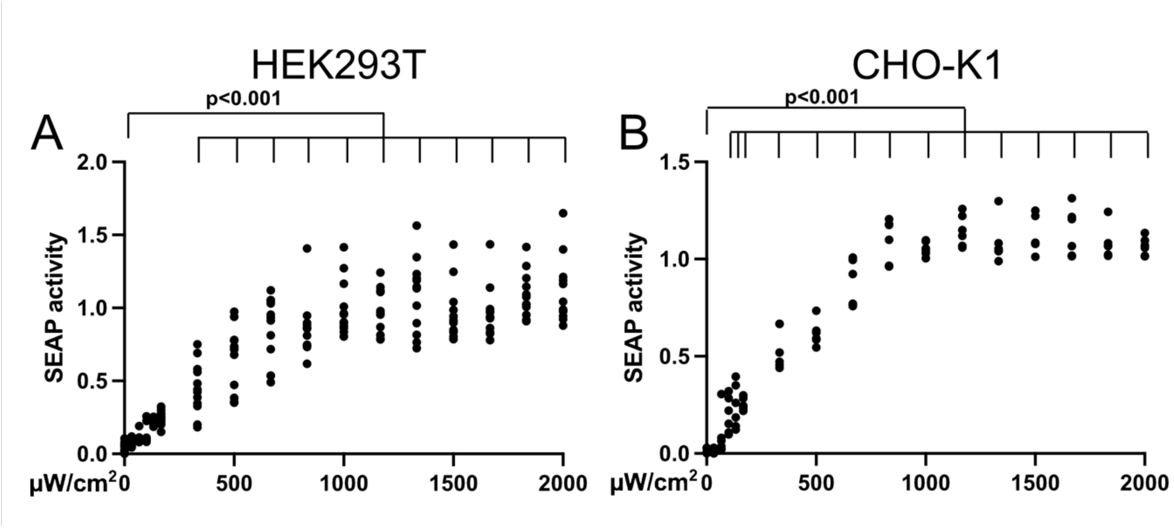

Supplementary Figure 5: Light-dose-dependent SEAP activity. Single values of dose-response curves of SEAP activity induced at indicated light intensities in A) HEK293T and B) CHO-K1 cells. Cells were excited 24 h after transfection for 24 h at indicated blue light intensities (always ON). The supernatant of these cells was added at a dilution of 1:100 on HEKblue IFN cells and incubated for 24 h. Values were normalized to CMV-driven SEAP activity of respective experiments. Mean ± SD (n = 6). Dots show means of independent triplicates from independent experiments. Statistical analysis was performed using one-way ANOVA and the Holm-Sidak post-hoc test.

Supplementary Figure 6

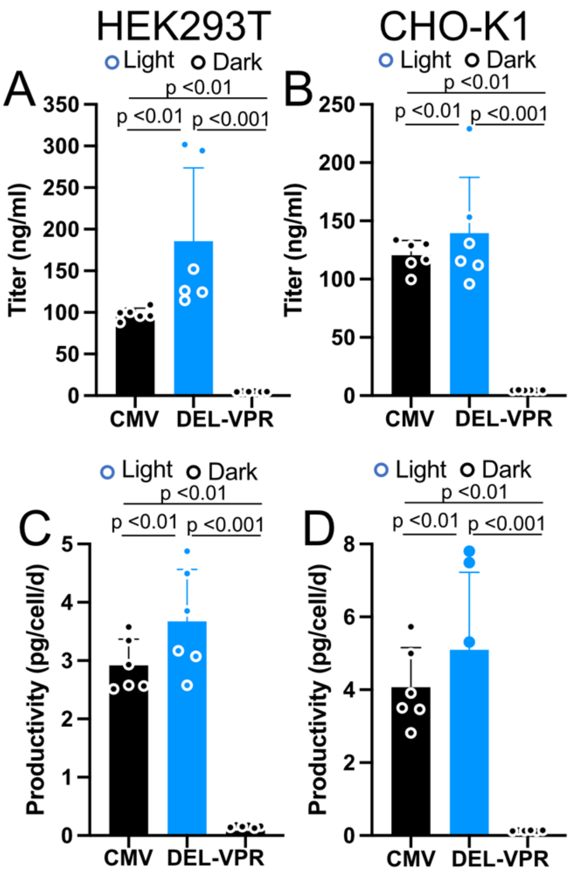

Supplementary Figure 6: Light-dependent titration of mAb expression in HEK293T and CHO-K1. A) and B) show the titer of mAb of the light-induced (DEL-VPR light; 1500 μW/cm^2), non-induced (DEL-VPR dark), or the constitutive (CMV) conditions after 24 h expression in HEK293T (A) or CHO-K1 (B), respectively, quantified by ELISA. C) and D) show the productivity of mAb production of the experiment presented in A-B) in HEK293T (C) and CHO-K1 (D), respectively. A-D) Means of biological independent samples ± SD (n = 6) are presented as bars, dots indicate individual values of each sample. Statistical analysis was performed using one-way ANOVA with the Holm-Sidak post-hoc test.

Supplementary Figure 7

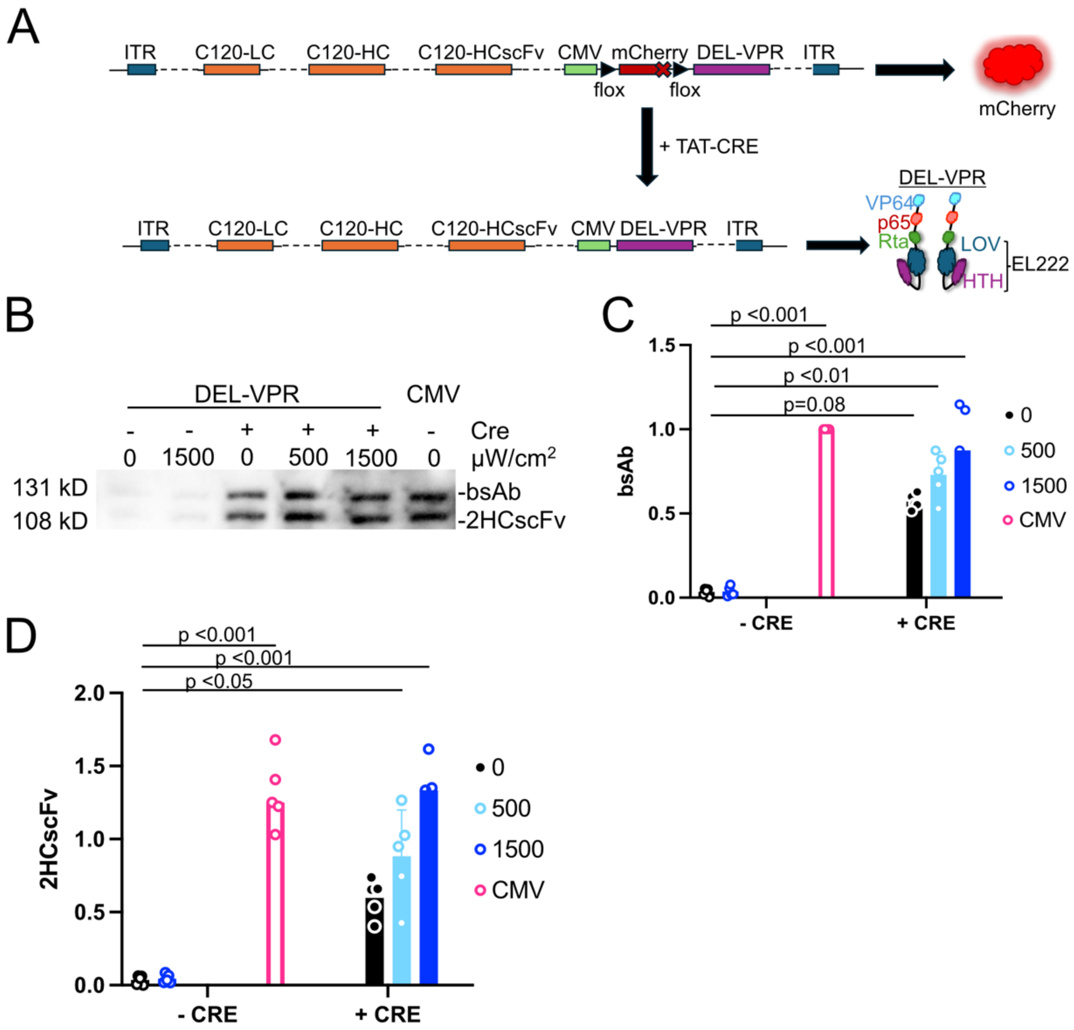

Supplementary Figure 7: Stable light-dependent titration of bsAb expression in HEK293T. A) The construct for piggyBac-mediated integration contains flanking inverted terminal repeats (ITR) at both ends. The 5'ITR is followed by C120-LC, C120-HC and C120-HCscFv. It also contains a CMV-driven floxed-mCherry with a stop codon followed by DEL-VPR. Under basal conditions, during the selection and expansion phase, only mCherry is expressed. Upon TAT-Cre treatment, mCherry gets cut out, including its stop codon, therefore, DEL-VPR is getting expressed and can induce the expression of the three bsAb chains. B) Western Blot of DEL-VPR-mediated expression in the non-activated (- Cre) and the activated (+ Cre) state with various blue light intensities to induce bsAb expression in stably integrated HEK293T cells. Quantification of the blots shown in (B) of the bsAb (C) and the HCscDv dimer (2HCscFv) (D). Data is normalized to bsAb intensity of the CMV control. (C-D) Mean $\pm$ SD (n = 3) are presented as bars, dots indicate individual values of each sample. Statistical analysis was performed using three-way ANOVA with the Holm-Sidak post-hoc test.

Supplementary Figure 8

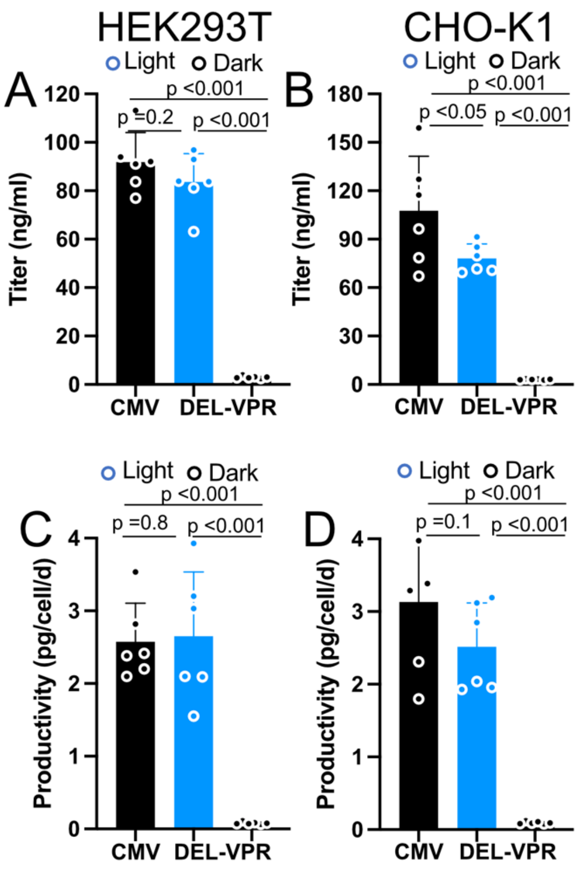

Supplementary 8: Light-dependent titration of bsAb expression in HEK293T and CHO-K1.

A) and B) show the titer of bsAb of the light-induced (DEL-VPR; 1500 µW/cm^2), non-induced (DEL-VPR) or the constitutive (CMV) conditions after 24 h expression in HEK293T or CHO-K1, respectively, quantified by ELISA. C) and D) show the productivity of bsAb production of the experiment presented in A-B) in HEK293T and CHO-K1, respectively. A-D) Means $\pm$ SD (n = 6) are presented as bars, dots indicate individual values of each sample. Statistical analysis was performed using one-way ANOVA with the Holm-Sidak post-hoc test.
